# The influence of the microbiota on immune development, chronic inflammation, and cancer in the context of aging

**DOI:** 10.15698/mic2019.08.685

**Published:** 2019-05-13

**Authors:** Taylor N. Tibbs, Lacey R. Lopez, Janelle C. Arthur

**Affiliations:** 1 Department of Microbiology and Immunology, University of North Carolina at Chapel Hill, Chapel Hill, North Carolina, United States of America.; 2 Center for Gastrointestinal Biology and Disease, University of North Carolina at Chapel Hill, Chapel Hill, North Carolina, United States of America.; 3 Lineberger Comprehensive Cancer Center, University of North Carolina at Chapel Hill, Chapel Hill, North Carolina, United States of America.

**Keywords:** microbiota, aging, chronic inflammation, cancer, immune development, immune maturation, microbiome, immunosenescence

## Abstract

From birth, the microbiota plays an essential role in human development by educating host immune responses. Proper maturation of the immune system perturbs chronic inflammation and the pathogenesis of disease by preventing inappropriate immune responses. While many have detailed the roles of specific microbial groups in immune development and human disease, it remains to be elucidated how the microbiota influences the immune system during aging. Furthermore, it is not yet understood how age-related changes to the microbiota and immune system influence the development of age-related diseases. In this review, we outline the role of the microbiota in immune system development as well as functional changes that occur to immune cell populations during immunosenescence. In addition, we highlight how commensal microbes influence the pathogenesis of cancer, a prominent disease of aging. The information provided herein suggests that age-related changes to the microbiota and immune system should be considered in disease treatment and prevention strategies.

## INTRODUCTION

Our microbiota is an integral part of our mammalian selves. Indeed, all multi-cellular eukaryotic hosts across the tree of life have an essential and characteristic microbiota that influences host development and resistance to disease. Complex organisms, such as vertebrates, host numerous microbial communities whose composition and function are relevant to their habitat at different body sites, such as the intestines (“gut”), skin, and oral cavity. The human gut microbiota is perhaps the best studied, most abundant, and arguably, the most influential microbiota that impacts host phenotypes [1]. In recent decades, the development of several scientific tools has exponentially increased our understanding of the microbiota and interactions with its human host. These include model organisms, most notably laboratory mice, that are born and raised germ-free (GF) and then colonized with known individual strains or groups of microbes – “gnotobiotic”. Through the use of GF and gnotobiotic mice, we have been able to demonstrate causality of specific microbes and microbial groups with distinct processes on immune development and non-infectious diseases like chronic inflammation and cancer, among others [1–3]. To validate the physiologic relevance of observations made in model organisms with human disease, we can now survey the human microbiota at an unprecedented depth using culture-independent molecular methods (i.e. targeted 16S “microbiome” sequencing, metagenomics, metatranscriptomics, and metabolomics) coupled with sophisticated bioinformatics pipelines. An important finding from population studies of the microbiome has revealed that the compositional fluctuations in an individual's microbiome over time are less substantial than inter-individual differences at a particular stage in development. However, the developmental changes that occur during early life and over an individual's lifespan certainly shape the composition and function of the microbiota [4]. On the other hand, the composition and functional capabilities of the microbiota shape host development [1]. In this review we discuss the current state of knowledge regarding the influence of our mammalian microbiota on the immune system, chronic inflammation, and a prominent disease of the aging – cancer.

## THE IMMUNE SYSTEM AND MICROBIOTA IN AGING

**Figure 1:**
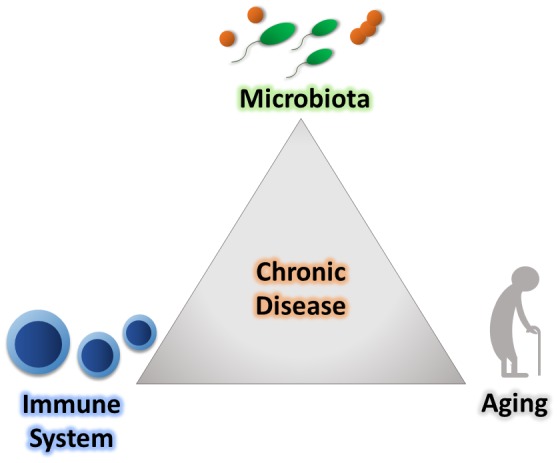
FIGURE 1: Aging, changes in immune response, and alterations of the microbiota contribute to chronic disease development. Relationships between the immune system, native microbiota, and general age-related biological changes all influence chronic inflammation and aging-related disease.

The immune system is our major host defense system that is educated early in life to distinguish harmful stimuli, including microorganisms. It is broadly subdivided into two branches, the innate and adaptive immune systems. The innate immune response is the first line of defense towards invading pathogens. For example, this branch of the immune system is involved when conserved pathogen-specific molecules trigger host cell pattern recognition receptors, which promotes an immediate and broad-spectrum protective response to pathogens. Conversely, the vertebrate adaptive immune response relies upon binding of unique antigens to specialized receptors, which ultimately leads to activation of T and B lymphocytes and the creation of pathogen-specific immunological memory. Through precise coordination, these systems provide host defense against foreign invaders. However, to efficiently mediate its response, the immune system must accurately distinguish between resident host-associated organisms and those that are potentially deleterious [5]. Over the course of our lifetime, our immune system encounters a diverse range of stimuli in a variety of contexts, which challenges the ability of the immune system to differentiate between self and non-self [6]. Alterations in this response can result in the development of a variety of diseases that include chronic inflammatory diseases, like inflammatory bowel disease (IBD) and cancer [7, 8]. As these immune responses are shaped over a lifetime, it indicates that age impacts the recognition of stimuli, which could transfer towards inappropriately reacting to residential host microbes. Inappropriate reactions to resident commensal bacteria may underlie a variety of pathogenic processes associated with aging **(Figure 1)**.

### Microbial influences on immune development

Establishment of the microbiota begins from birth and continues until post-weaning [9]. During establishment, the microbiota is highly diverse and prone to fluctuations based on environmental and dietary changes [10]. After 2-3 years of age, this complex community stabilizes with the majority of bacterial community members remaining unchanged throughout an individual's lifespan [11]. After stabilization, the core human microbiota mainly comprises the following phyla: Bacteroidetes and Firmicutes, with a smaller abundance of Actinobacteria, Proteobacteria, and Verrucomicrobia [12]. The aging process strongly impacts the composition of the microbiota as individuals with increased frailty, an assessment of biological age based on current health status and life expectancy, lose bacterial diversity and form a Bacteroidetes dominant population [13]. This loss of microbial diversity may potentiate aging and disease. Indeed, an aging model in turquoise killifish reported that older fish had a marked decrease in gut microbial diversity which favored more pathogenic genera [14]. Moreover, transferring the gut microbiota of these young turquoise killifish into middle-aged fish helped retain species diversity and significantly increased overall lifespan [14]. While not directly correlated to chronological aging, this loss of bacterial diversity most often begins in humans between 75-80 years of age and is a form of dysbiosis that potentiates disease development [15]. These results are a generality derived from a diverse population, and only consider numerical age (lifespan) rather than the individual's aging-related health status (healthspan) [16]. Despite this correlation, it is important to remember that the microbiota play a largely protective role in disease development by initiating and educating the host immune system [17].

Early observations in GF mice demonstrated that the host microbiota is essential for the maturation of the immune system [17, 18]. In the absence of a microbiota, GF mice have several immunological defects, including reduced lymphoid cell numbers and function [19]. For example, GF mice have fewer T helper type 1 (Th1) cells compared to their conventionalized counterparts [20]. Th1 cells promote cell-mediated immune responses and phagocyte-dependent inflammation to target intracellular pathogens [21]. Th1 responses in GF mice can be restored through host colonization with a variety of microbes, including the well-studied pathogen *Listeria monocytogenes,* which promotes Th1 development through macrophage production of the T cell-stimulating factor, interleukin 12 (IL-12) [22]. Intracellular bacteria like *L. monocytogenes* specifically induce Th1 responses in the gut [23]. Additionally, GF mice have a reduced number of T helper type 17 (Th17) cells. Th17 cells are generally pro-inflammatory, however, they drive production of IL-17 and mediate defense against extracellular pathogens and autoimmune disease [20, 21]. Adherent bacteria, such as Clostridia-related segmented filamentous bacteria (SFB), induce the development of Th17 cells in the small intestine by driving the release of serum amyloid A from intestinal epithelial cells (IECs). The release of serum amyloid A results in the production of innate lymphoid cell group 3 (ILC3) cytokines which upregulate the Th17 response [24]. Fine-tuning of Th1 and Th17 responses are essential for immune tolerance towards the host microbiota, as seen in the case of IBD where aberrant populations of Th1 and Th17 cells lead to enhanced pathology [7]. Underdevelopment of these responses may underlie the progression of other diseases associated with chronic inflammation, such as cancer [25, 26].

The absence of a microbiota impacts most, if not all, aspects of the immune system [1]. However, we are just beginning to understand precisely which microbes induce specific effects, and where the window of opportunity lies for correcting many of these immune deficiencies. One study examining colonic invariant natural killer T (iNKT) cell populations revealed that this opportunity for modulation likely occurs during infancy, prior to weaning [27]. At birth, GF mice have an enriched population of colonic lamina propria iNKT cells compared to specific-pathogen-free (SPF) mice [28]. iNKT cells are pro-inflammatory and mediate tolerance to commensal microbes [29]. Colonization of adult (>5 weeks of age) GF mice with a complex microbiota does not influence the number or activity of iNKT cells [27]. However, if the colonization occurs when GF mice are neonates, the number of iNKT cells is reduced and their later activation is well-controlled [27]. This early education of the colonic iNKT cell population is important for limiting morbidity associated with IBD [27]. This supports the idea that exposure to specific microbes and microbial products is needed within a certain developmental time for the host to appropriately educate target immune populations and prevent disease.

### The immune system and aging

The presence of a microbiota in early life is essential for immune system maturation. However, education of the immune response is a lifelong process. Alterations to the innate and adaptive immune systems which occur with increased frailty are linked to a complex biological process known as immunosenescence [30]. Specific changes associated with immunosenescence can best be understood through functional differences within the unique cell types of the innate and adaptive immune systems. For cells of the innate immune system, there are reported functional differences for every major cell type [31]. However, the most distinct differences are within neutrophil and macrophage populations. Neutrophils isolated from the blood of individuals (aged 62-83 years old) displayed reduced phagocytic capabilities and decreased production of reactive oxygen species (ROS) when infected with *Staphylococcus aureus*, which correlated with impaired bactericidal activity [32]. Neutrophils are the first line of defense towards invading pathogens; therefore, immunosenescence-related changes to this cell type suggest an age-related decline in pathogen-induced responses and tolerance towards resident microbes [5]. Similarly, primary macrophages isolated from aged mice (18-24 months old) exhibit impaired phagocytosis and reduced ROS production in response to infection, when compared to macrophages isolated from young mice (2-3 months old) [33, 34]. Additionally, macrophages from aged mice displayed modifications in antigen presentation and reduced production of pro-inflammatory cytokines [35, 36]. Alterations in macrophage antigen presentation and cytokine release may lead to defective immune signaling between the innate and adaptive immune systems, resulting in a weakened immune response [5]. Overall, age-related changes to the innate immune system strongly reduce the host's initial response to pathogens and how the innate system informs adaptive responses. Strategic communication and coordination between these systems is required for proper immune functioning; therefore, maladaptive innate immune responses can misinform the subsequent adaptive immune responses and contribute to the development of disease.

While changes in the innate immune system have been noted with aging, long-term effects of the microbiota on adaptive responses are more pronounced [31, 37]. The adaptive immune system is used for long-term protection from environmental insult and invading pathogens. Therefore, long-term education of this subsystem could have additive effects on immunosenescence. B cells are a major cell type of the adaptive immune system. The population of antigen-experienced B cells is divided into plasma cells and memory B cells. Plasma cells produce pathogen specific antibodies, while memory B cells provide long-term recognition of antigens via their ability to quickly reactivate upon subsequent antigen encounter [38]. Peripheral blood isolated from elderly individuals (aged 86-94 years old) showed a reduction in B cell population diversity that was attributed to a decrease in memory B cells [39]. This decline in B cell diversity was linked to increased frailty and could be used as a predictor for general health status [39]. A reduction in memory B cell numbers may cause an inappropriate immune response towards the microbiota, as B cells are important for establishing the distinction between pathogenic and commensal bacteria [38]. The reduction in memory B cell numbers that accompanies age may facilitate inappropriate immune responses towards the microbiota, promoting microbial dysbiosis and enhancing disease risk.

T cells are the second major cell type of the adaptive immune system and are classified as either conventional or unconventional T lymphocytes [40]. This classification is based on unique T cell surface markers, functional ability, and body site localization [40]. In general, T cells become activated upon binding of an antigen which is displayed on the surface of antigen presenting cells (APC) [41]. Once activated, conventional T cells can perform a wide range of functions from promoting long-term immunity to killing infected cells [41]. During immunosenescence, one of the most prominent changes to occur within conventional T cell populations is the reduction of CD28^+^ T cells [42]. CD28 is a co-stimulatory protein expressed on naïve T cells and is important for T cell activation, regulation, and survival. Therefore, a reduction in CD28^+^ T cells may lessen T cell activation causing increased susceptibility to pathogens [43]. Additionally, the loss of CD28 may decrease tolerance to self-antigens and the microbiota as it is also a negative regulator of immune responses [43, 44]. To highlight this point, a recent article revealed an age-related reduction in naïve CD8^+^ T cells, which could compromise host response to pathogens and self-antigens [45]. Conversely, this group found an age-associated increase in memory CD4^+^ T cells, which corresponds to the cumulative effects of a lifelong antigenic load [45]. Alterations in conventional T cell populations may contribute to chronic inflammation and the onset of age-related disease thorough inappropriate immune responses towards pathogens and the self.

While conventional T cells perform a wide range of functions, their role during immunosenescence is complex and not fully detailed. On the other hand, a class of unconventional T cells, known as natural killer T (NKT) cells, are strongly influenced by the microbiota and immunosenescence. In two separate studies, populations of T cells isolated from the peripheral blood of elderly patients showed a reduction in the proportion of NKT cells versus cells isolated from young patients [46, 47]. Additionally, NKT cells isolated from the liver of aged mice (aged >20 months old) demonstrated a decline in cytotoxic effector function, and reduced cytokine release versus NKT cells isolated from young mice (aged 2 months old) [48]. This decline in NKT cell number and immunological function may exacerbate disease development by weakening the host's response to pathogens and reducing immunotolerance towards the microbiota.

A decrease in the proportion of CD28^+^ T cells and NKT cells may potentiate the development of autoimmune diseases within elderly populations by reducing tolerance to self-antigens. However, despite an increase in autoantigens within aging individuals, old-age is not a major risk factor for most autoimmune diseases. Studies looking at the proportion of regulatory T cells (Tregs), demonstrate that the repertoire of peripheral Tregs is higher within elderly humans [49]. Since Tregs promote tolerance to self-antigens, the higher proportion of Tregs in aging individuals could be working against perturbations in immunocompetence to prevent autoimmune diseases. More studies are needed to demonstrate age-related changes in Treg functional capacity. Nevertheless, an increased proportion of Tregs is not without some cost as immunosuppression by Tregs may promote chronic infections, reduce vaccine efficiency, and increase rates of cancer among the elderly. However, it remains to be seen what impact the microbiota has on T cell-based immunosenescence in the context of aging.

The host microbiota initiates immune system maturation in early life. However, to keep up with a lifelong antigenic load, the immune response must be fine-tuned and properly educated across the lifespan. Despite these observations, it remains unclear how age-related immunological changes impact cellular crosstalk and overall immunocompetence. On top of this, how the microbiota impacts the immune system during immunosenescence remains to be elucidated. It is likely that changes to the immune system result in an inappropriate response towards commensal microbes, as indicated by diseases like IBD [3, 50]. Inappropriate reactions to the native microbiota and lessened ability to control invading pathogens may contribute to the development of chronic inflammation and the onset of age-related diseases, such as cancer [51].

## MICROBIOTA AND CANCER – A DISEASE OF AGING

Cancer is considered a disease of old age. As life expectancy increases, the estimated rate of cancer is predicted to increase by 45% from 2011 to 2030 in the United States [52]. It is also estimated that by 2030, individuals 65 years and older will contribute to 70% of all cancers in the U.S. [53]. The risk of developing cancer increases dramatically with age as the duration of time in which an individual is exposed to carcinogens increases [54], the proliferative capacity of aging cells decreases [55], and immunological competence decreases [56]. Cancer typically results from a series of genetic mutations or epigenetic modifications that develop sequentially overtime [57]. The colon, which harbors the largest and most diverse microbiota of all organs, has the highest incidence rate of all reported cancers in the 85+ population [58]. Over the past decade, we have become increasingly aware of the roles that the microbiota play in the development of cancer and modulation of cancer therapies. We have also elucidated several mechanisms underlying the microbial influences on cancer. However, these roles are diverse and seem to influence many aspects of immune and cancer development [59]. Microbes can contribute to the onset and progression of cancer through direct means, such as by producing genotoxins, and indirect means through the modulation of immune responses to tumors and immunotherapy [60–64]. Additionally, several members of the native microbiota can alter chemotherapeutic drugs, resulting in morbid side effects for the host or even rendering them clinically inert [65, 66]. It is therefore important to divulge the significance of microbial interactions on age-related diseases, such as cancer, in order to fully understand disease progression and design suitable therapies. Here we will discuss known mechanisms by which the microbiota can influence the onset of cancer, endogenous anti-cancer immune responses, chemotherapeutic activities, and anti-tumor immunotherapy.

### Endogenous anti-cancer immune responses

The ability to manipulate the microbiota using GF and gnotobiotic mice has demonstrated the importance of the microbiota on immune system development and the gastrointestinal environment. A notable example is microbial modulation of bile acid composition, which can influence immune responses and also affect the development of malignancies. Host-derived primary bile acids are converted into secondary bile acids by the gut microbiota, primarily by members of the genus *Clostridia,* and circulated systemically throughout the body via hepatic circulation [67]. Previous work illustrated that secondary bile acids can increase the risk of obesity-associated hepatocellular carcinoma in susceptible mice [68]. Recent data suggests that antibiotic elimination of the gut microbiota in mice decreases both primary and metastatic tumors within the liver by facilitating the buildup of primary bile acids, which trigger liver-specific NKT cell recruitment to target cancer cells [69]. However, the influences of *Clostridia spp.* on the development of cancer are likely more complex. Treatment of colorectal cancer (CRC)-prone mice with the probiotic cocktail VSL#3, a mixture of lactic acid-producing bacteria with anti-inflammatory properties, decreased the population of *Clostridia spp.* in the gut and subsequently enhanced tumorigenesis, suggesting that some *Clostridia spp.* may also be protective against the onset of malignancies [70]. These data highlight how further understanding the conditions by which particular species promote or perturb the development of cancer must be addressed when assessing cancer risk, prevention, and treatment. The profound effects the bacteria elicit on cytotoxic immune cells and tumor development provide key insights on how the native microbiota influence host anti-cancer responses.

*Fusobacterium nucleatum*, a Gram-negative oral commensal overrepresented in CRC, can promote tumorigenesis via direct effects on the epithelium and through the modulation of endogenous immune responses [62, 71]. A known target is the natural killer (NK) cell, which kills compromised host cells, such as infected or cancerous cells. *F. nucleatum* inhibits the cytotoxicity of NK cells via the Fusobacterium protein Fap2, which binds the NK cell inhibitor receptor TIGIT (T cell immunoglobulin and ITIM or immunoreceptor tyrosine-based inhibition motif domain) [72]. In addition to targeting the immune system, *F. nucleatum* exerts procarcinogenic activities directly on epithelial cells through β-catenin signaling, altering proliferation and cell fate [73]*. F. nucleatum* can also alter the efficacy of chemotherapeutic drugs by inhibiting host cell apoptotic pathways [74]. *F. nucleatum* is a prime example of one species of the microbiota that exhibits a variety of different effects on the host to mediate tumorigenesis and hinder cancer therapy. In the next few sections, we will discuss a variety of known bacterial mechanisms that act upon cancer development and treatment.

### Cancer immunotherapy, the microbiota, and aging

Several independent groups have recently demonstrated that some members of the microbiota play critical roles in determining patient responsiveness to cancer immunotherapy. The exact mechanisms by which individual species of bacteria exhibit these effects are not fully understood. However, current data suggest that bacterial modulation of the immune system may be one critical mode of altering host response to cancer therapy. Recent data regarding anti-PD1 therapy supports this notion. Anti-PD1 treatment is a type of immune checkpoint inhibitor that enhances anti-tumor immune responses by maintaining T cell activation via blocking the immune inhibitory receptors programmed death ligand-1 and 2 (PDL-1 and PDL-2) [75]. Anti-PD1 therapy is often prescribed to patients with lung cancer and advanced melanoma. However, the efficacy of anti-PD1 immunotherapy ranges from only 19 to 43% for both cancer types [76, 77]. Several members of the microbiota are enriched in PD-1 responders, including *Bifidobacterium longum, Collinsella aerofaciens*, and *Enterococcus faecium* [78]. *Faecalibacterium,* an abundant Gram-positive genus of commensals in the human gut, was also enriched in PD1-responders [61]. Tumor-bearing mice that were given fecal microbiota transplants (FMTs) from PD1-responders exhibited decreased tumor burden and tumor size when receiving anti-PD1 therapy. *Faecalibacterium* promoted cytotoxic (CD8+) T cell recruitment to tumors, which may be an important mechanism underlying the ability of this bacterial group to enhance anti-PD1 responses and reduce tumor burden [61]. Similarly, FMTs from PD-1 responders enhanced PD-1 treatment in recipient mice, which was further augmented with oral supplementation of the commensal *Akkermansia muciniphila* [79]. Antibiotic treatment reduced the efficacy of PD-1 immunotherapy in mice, consistent with clinical reports of reduced PD-1 efficacy in patients simultaneously taking antibiotics [79]. These studies demonstrate that multiple species of bacteria have the capability of altering immunotherapeutic responses in patients. Moving forward, it will be critical to consider the contributions of these microbial communities when developing anti-tumor immunotherapies.

There is a paucity of data evaluating the combined influences of the microbiota and age in immunotherapeutic outcomes. Some studies have investigated the effects of age on immunotherapy; however, the majority of studies heavily rely on metadata and have found few differences in immunotherapeutic efficacy in relation to age [80]. One metadata study reported improved overall survival from anti-CTL4 treatment, but not anti-PD1 treatment in individuals > 75 years of age [81]. Another study in mice showed that CD40/IL-2 treatment for metastatic renal cell carcinoma increases mortality in aged mice compared to young mice, due to multi-organ failure/systematic toxicity [82]. This study illustrates that immunotherapies, which are commonly developed in young mouse models (2-4 months old), may not take into account the immune changes that occur in aging populations; therefore, the altered immune environment associated with aging should be considered when developing suitable immunotherapeutic strategies. Furthermore, side effects of immunotherapies may be exacerbated in the elderly due to other age-related deficiencies, such as increased risk of dehydration from reduced kidney function. The contribution of the microbiota on immunotherapeutic outcomes specifically in aged individuals is unknown. Clinicians should be thoughtful in prescribing immunotherapies to aged individuals and consider immunocompetency changes and individual microbial diversity that may exacerbate side effects or affect the efficacy of immunotherapeutic drugs.

### Chemotherapy and the microbiota

Multiple members of the microbiota can differentially influence cancer chemotherapy, with some enhancing and some inhibiting the clinical effects of chemotherapeutic drugs. An important early observation was that genotoxic platinum chemotherapies, including oxaliplatin and cisplatin, were ineffective in tumor-bearing GF mice, indicating that the presence of a complex microbiota is essential for these chemotherapies [83]. Platinum chemotherapies promote ROS to induce cytotoxicity. The DNA damage incited by cisplatin is augmented via the production of mitochondrial ROS within tumor-associated inflammatory cells and cancer cells themselves [83, 84]. It may be that in the absence of a native microbiota, inflammatory cells are not effectively primed to produce ROS during development, leading to shortcomings in ROS production later in life that may affect the efficacy of platinum-based chemotherapies. These data illustrate that a properly developed immune system trained by the native microbiota augment anti-tumor responses during chemotherapeutic treatment. Furthermore, these insights highlight the influential capacity of the microbiota on the host and how in their absence, the immune system may have substantial deficits in anti-tumor responses.

Conversely, the microbiota can have negative effects on chemotherapeutic efficacy. Deep sequencing for microbes within pancreatic tumor biopsies revealed that 57.5% of pancreatic tumor tissues tested (65 of 113 samples) were positive for bacterial reads, with Gammaproteobacteria being the most abundant (51.7% of reads) [66]. Interestingly, 98.4% of Gammaproteobacteria contain genes that encode a specific isoform of the enzyme cytidine deaminase (CDD_L_), which has the ability to break down gemcitabine and confer chemotherapeutic resistance in tumor tissues [66]. Accordingly, bacterial migration from the gastrointestinal tract into the pancreatic ducts and tumor tissue may be a significant source of drug failure in clinical pancreatic cancer cases. Gut bacteria are also responsible for re-activating chemotherapeutic drugs in the distal intestine. Irinotecan, a chemotherapeutic drug used to treat CRC, is inactivated by the liver, but reactivated into the active drug by *Clostridia spp.* through bacterial β-glucuronidases in the gut [85]. This re-activation in the distal intestine contributes to the typical morbid gastrointestinal side effects of irinotecan therapy, including mucositis and diarrhea [65, 85]. This evidence illustrates the profound impact the native microbiota can have on the response to cancer therapies. Therefore, future treatment plans should account for the influence of these patient-specific microbial factors to ensure successful chemotherapeutic outcomes.

### Direct effects of the microbiota on tumorigenesis

Specific members of the microbiota have the capacity to directly contribute to tumorigenesis [2, 59]. Commensal Enterobacteriaceae, including several strains of *Escherichia coli,* are capable of inducing DNA damage in mammalian cells by producing a genotoxin termed colibactin [60, 86]. The bacterial polyketide synthase (*pks*) pathogenicity island encoding colibactin is upregulated in CRC mouse models and the presence of these gene products promotes tumorigenesis by inducing double-stranded DNA breaks [87–89]. Colibactin can also induce premature cellular senescence in cells that initially survive the DNA damage [90, 91]. Furthermore, the *pks* pathogenicity island is overrepresented in the microbiota of CRC and IBD patients, who represent a population at high risk of developing CRC [60, 92, 93]. In another population at high risk for CRC, familial adenomatous polyposis patients, *pks+* bacteria are found in combination with other pro-carcinogenic microbes in colonic biofilms [94]. This suggests that bacterial genotoxins contribute substantially to the risk and development of chronic inflammatory diseases and human cancer.

Gut bacteria also have the capacity to induce a pro-tumorigenic environment through chronic inflammation. Enterotoxigenic *Bacteroides fragilis,* a member of the most abundantly represented genus in the gut, produces its own flavor of toxin called *B. fragilis*-derived toxin (BFT) [95]. BFT is a zinc-dependent metalloprotease that can induce colitis and promote tumorigenesis through the generation of ROS and subsequent initiation of DNA damage in epithelial cells [96]. Enterotoxigenic *B. fragilis* robustly activates Th17 immune responses, which involves the inflammatory cytokine IL-17, and may lower host anti-tumor immune responses, encouraging unhindered tumor growth [97, 98]. Enterotoxigenic *B. fragilis* is overrepresented in patients with CRC when compared to healthy individuals [64] and exacerbates tumorigenesis in susceptible mice [98]. Interestingly, the tumorigenic effects of *pks+ E. coli* and enterotoxigenic *B. fragilis* act synergistically *in vivo* to quicken tumor onset and increase mortality in susceptible mice beyond the capability that either species has individually [94]. Given that the native microbial community is quite complex, the cumulative effects of microbial products on the host may significantly contribute to the onset and progression of cancer.

While widely considered a pathogen, *Helicobacter pylori* is estimated to be present in the gastrointestinal tract of over half of the human population worldwide and a major risk factor for gastric adenocarcinoma [99, 100]. *H. pylori* was one of the earliest identified microbial suspects of inflammation-mediated cancer development and it is estimated that *H. pylori* infection increases the attributable risk of gastric cancer by 73% [101]. Chronic *H. pylori* infection results in inflammation and tissue damage by the bacterial virulence factor CagA (cytotoxin-associated gene A), which initiates the development of the hallmark precursory lesions of gastric cancer, including intestinal metaplasia and dysplasia [102]. It remains unclear why *H. pylori* infection only progresses to malignancy in a subset of infected individuals; however, it is postulated that host immune responses and the genetics of both host and microbiota contribute to neoplastic development [103].

**Figure 2:**
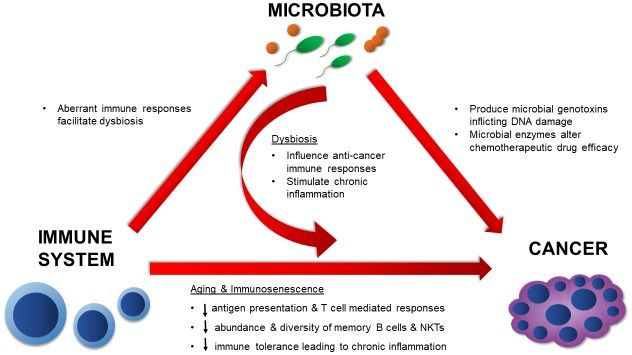
FIGURE 2: Interplay of the microbiota and immune system influence cancer development during aging. The immune system and native microbiota contribute to cancer both directly and indirectly via chronic inflammation, aberrant changes in various immune responses, DNA damage, and alterations to the efficacy of anti-cancer therapies.

In summary, the mechanisms by which the native microbiota influences cancer development and therapy are numerous and diverse **(Figure 2)**. The evidence presented here illustrates the diverse microbial mechanisms that contribute to tumorigenesis, whether that be by directly targeting the DNA for damage through a toxin or by providing an augmented environment for unrestricted cellular proliferation. Microbial effects on the immune system are undoubtedly involved in these processes. As more data surfaces, it will be imperative to synthesize and apply knowledge on positive and negative microbial contributions towards cancer development and treatment. By doing so, we can more effectively assess cancer risk and ultimately design more potent anti-cancer therapies.

## CONCLUSION

Our microbiota plays a central role in human health by educating our immune responses to recognize self versus non-self, across the lifespan. Immune system development begins at birth, with the introduction of the microbiota, and only fully matures in the presence of commensal microbial flora. Proper maturation of the immune system is necessary to prevent aberrant immune responses, which can lead to chronic inflammation and the onset of disease. It is well understood that fine-tuning the immune response is a lifelong educational process; therefore, it is important to consider how the microbiota and immune system change throughout aging. While studies in this review focus on changes linked to chronological age, it is necessary to consider how biological age (assessed by health status and life expectancy) shapes the microbiota and immune system. As we highlight here, a reduction in microbial diversity is linked to increased frailty. Additionally, all major cell types of the innate and adaptive immune systems are functionally altered in the context of aging by a process known as immunosenescence. Despite these marked differences, not much is known about the connection between the microbiota and immune-senescence. Traditionally, studies have focused on classic pathogens and the burden of a lifelong antigenic load as they relate to the improvement of vaccine efficiency in the elderly. However, decreased ability to fight off foreign pathogens is not the only concern of aging. In fact, biological age puts elderly at risk for a wide range of age-related diseases, including cancer, cardiovascular disease, and Alzheimer's disease, all of which have been shown to be influenced by the microbiota [2, 3, 104–108] While in this review we focus upon how the microbiota and immune system influence the pathogenesis of cancer, it is worth considering the other changes that occur physiologically with aging, how this impacts our microbiota, and vice versa. There is no universal microbiota composition known to mediate inflammation or anti-tumor responses. This is because the microbiota and immune system are unique to each individual and cultivated over a lifespan. Therefore, biological age should be considered in pre-clinical models, as age-related factors will likely affect therapeutic efficacy and outcomes. Age-related changes influence both the host and microbiota; therefore, they should be considered during the design of animal and human studies to provide a more holistic understanding of disease treatment and prevention strategies.

## Abbreviations:

CRC: – colorectal cancer,
GF: – germ-free,
IBD: – inflammatory bowel disease,
IL: – interleukin,
iNKT: – invariant natural killer T,
NK: – natural killer,
NKT: – natural killer T,
ROS: – reactive oxygen species,
Th: – T helper,
Treg: – regulatory T cell.
